# Natural T‐cell ligands that are created by genetic variants can be transferred between cells by extracellular vesicles

**DOI:** 10.1002/eji.201747152

**Published:** 2018-08-17

**Authors:** Anita N. Kremer, Marijke I. Zonneveld, Andreas E. Kremer, Edith D. van der Meijden, J.H. Frederik Falkenburg, Marca H.M. Wauben, Esther N.M. Nolte‐‘t Hoen, Marieke Griffioen

**Affiliations:** ^1^ Department of Hematology Leiden University Medical Center Leiden The Netherlands; ^2^ Department of Internal Medicine 5 Hematology and Internal Oncology University Hospital Erlangen Erlangen Germany; ^3^ Division of Pharmacology Department of Pharmaceutical Sciences Faculty of Science Utrecht University Utrecht The Netherlands; ^4^ Department of Biochemistry & Cell Biology Faculty of Veterinary Medicine Utrecht University Utrecht The Netherlands; ^5^ Tytgat Institute for Liver and Intestinal Research Academic Medical Center University of Amsterdam Amsterdam The Netherlands; ^6^ Department of Internal Medicine 1 Friedrich‐Alexander‐University of Erlangen‐Nuremberg Erlangen Germany

**Keywords:** Antigens, Antigen presentation, Extracellular vesicles, T cells, Tumor Immunity

## Abstract

CD4 T cells play a central role as helper cells in adaptive immunity. Presentation of exogenous antigens in MHC class II by professional antigen‐presenting cells is a crucial step in induction of specific CD4 T cells in adaptive immune responses. For efficient induction of immunity against intracellular threats such as viruses or malignant transformations, antigens from HLA class II‐negative infected or transformed cells need to be transferred to surrounding antigen‐presenting cells to allow efficient priming of naive CD4 T cells. Here we show indirect antigen presentation for a subset of natural HLA class II ligands that are created by genetic variants and demonstrated that (neo)antigens can be transferred between cells by extracellular vesicles. Intercellular transfer by extracellular vesicles was not dependent on the T‐cell epitope, but rather on characteristics of the full‐length protein. This mechanism of (neo)antigen transfer from HLA class II‐negative cells to surrounding antigen‐presenting cells may play a crucial role in induction of anti‐tumor immunity.

## Introduction

CD4 T cells play a central role as helper cells in adaptive immunity 1. MHC class II positive macrophages, B‐cells and dendritic cells are well equipped to take up, process and present exogenous antigens in their MHC class II molecules, and priming of naive CD4 T cells by these professional antigen presenting cells (APC) is a crucial step in efficient induction of immune responses 2. Upon recognition of epitopes in MHC class II molecules, antigen‐specific CD4 T cells become activated and secrete cytokines, thereby providing help to induction and maintenance of CD8 cytotoxic T‐lymphocytes 1, 3, stimulation of B‐cells to produce antibodies 4, maturation of dendritic cells 1 and induction of delayed type hypersensitivity reactions by activation of macrophages 4, 5. As such, CD4 T‐helper cells are crucial in eliciting potent immunity against exogenous pathogens.

For efficient induction of immunity against intracellular threats, such as viruses or malignant transformations, intracellular antigens from HLA class II‐negative infected or transformed cells need to be transferred to surrounding APC in order to prime naive CD4 T cells. This concept of antigen transfer is well known from solid organ transplantation where presentation of donor antigens on recipient APC has been linked to chronic rejection 6, 7. Also in mice after MHC‐matched allogeneic bone marrow transplantation, donor APC presenting recipient antigens have been shown to stimulate CD4 T cells and mediate Graft‐versus‐Host disease 8. A similar mechanism in which CD4 T‐helper cells are primed by APC presenting differentiation antigens taken up from highly specialized HLA class II‐negative tissues may explain the predisposition of human individuals with specific HLA class II alleles to develop autoimmune diseases 9. Finally, in tumor immunology, indirect presentation of tumor‐associated antigens by professional APC has been shown to induce beneficial immune responses in which MHC class II‐negative tumors can be rejected in a CD4 T‐cell dependent fashion 10, 11, 12.

In addition to tumor‐associated antigens, which are self‐proteins that are overexpressed in tumors, HLA ligands created by genetic variants can be recognized by the immune system and lead to potent anti‐tumor responses. These non‐self or neoantigens can be encoded by genetic polymorphisms that are targeted by donor T cells after allogeneic stem cell transplantation 13, 14, 15 or by somatic mutations that are recognized by autologous T cells in non‐transplanted cancer patients 16, 17. Remarkably, HLA class II‐restricted neoantigens have been identified as T‐cell targets in patients with HLA class II‐negative solid tumors 17, strongly suggesting that also neoantigens can be transferred from tumor cells to HLA class II‐positive APC for induction of specific CD4 T cells. We here show indirect antigen presentation for a subset of natural HLA class II ligands that are created by genetic variants and demonstrate that these antigens can be transferred between cells by full‐length proteins that are secreted in extracellular vesicles (EV). This mechanism of (neo)antigen transfer from HLA class II‐negative tumor cells to surrounding APC via EV may play a crucial role in induction of anti‐tumor immunity.

## Results

### Intercellular transfer of natural HLA class II ligands that are created by genetic variants

To investigate the occurrence of indirect antigen presentation, we selected 6 HLA class II ligands encoded by genetic polymorphisms that are targeted by donor CD4 T cells after allogeneic hematopoietic stem cell transplantation. The antigens were derived from cytosolic kinases (PTK2B and PI4K2B), membrane proteins (LY75 and MR1), cytosolic enzyme (MTHFD1) and nuclear/cytosolic RNA helicase (DBY). The antigens were restricted by different HLA‐DR and ‐DQ alleles: DRB1*03:01/A*01:02 (MTHFD1), DRB1*13:01/A*01:02 (LY75), DRB3*01:01/A*01:02 (PTK2B), DRB3*02:02/A*01:02 (MR1), DQB1*06:03/A*01:03 (PI4K2B) and DQB1*05:01/A*01:01 (DBY). Five proteins (LY75, MR1, PTK2B, PI4K2B and MTHFD1) were identified as minor histocompatibility antigens targeted by specific CD4 T cells in a female patient after allogeneic stem cell transplantation with her HLA‐matched sister 14, 15, whereas the remaining protein (DBY) is encoded on the Y‐chromosome and recognized by CD4 T cells in a male patient after allogeneic stem cell transplantation with his HLA‐identical sister 18.

**Figure 1:**
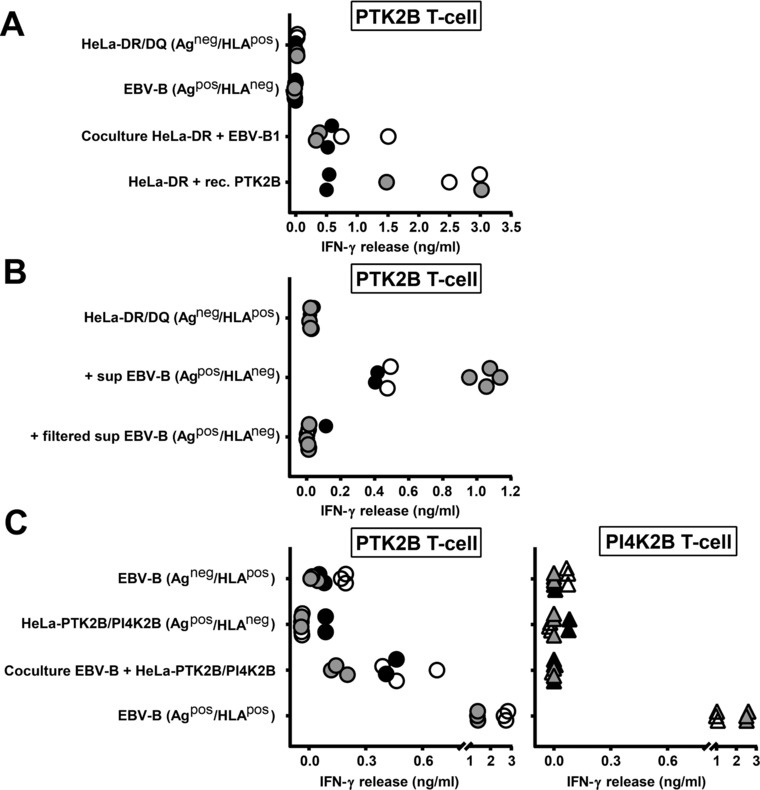
Intercellular transfer of the natural HLA class II ligand of PTK2B. (A) EBV‐B cells endogenously expressing the PTK2B antigen, but lacking the relevant HLA‐DRB3*01:01/A*01:02 restriction allele (Ag^pos^/HLA^neg^ donor cells) were co‐cultured with antigen‐negative HeLa cells that were transduced with HLA‐DRB3*01:01/A*01:02 (Ag^neg^/HLA^pos^ acceptor cells). After overnight coculture, PTK2B‐specific T cells were added and IFN‐γ release was measured by ELISA. As positive control, HLA class II‐transduced HeLa cells were exogenously loaded with recombinant PTK2B. Results of duplicate wells pooled from three independent experiments represented by open, gray and black dots are shown. (B) Culture supernatants from antigen‐positive EBV‐B donor cells lacking the relevant HLA‐DRB3*01:01/A*01:02 restriction allele were centrifuged at 300 × *g* to remove viable cells and cell debris, and loaded on antigen‐negative HeLa cells transduced with HLA‐DRB3*01:01/A*01:02. In addition, culture supernatants were passed through filters to remove proteins and particles with sizes >10, 30 or 100 kDa, and flow throughs were loaded on HeLa acceptor cells. Antigen uptake, processing and presentation into HLA class II was tested by measuring recognition by PTK2B‐specific T cells in IFN‐γ ELISA. Results of duplicate or quadruplicate wells pooled from three independent experiments represented by open, gray and black dots are shown for culture supernatants and flow throughs of 30 kDa filters. (C) HLA class II‐negative HeLa cells transduced with wild‐type PTK2B or PI4K2B (Ag^pos^/HLA^neg^ donor cells) were cocultured with antigen‐negative EBV‐B cells endogenously expressing the HLA‐DRB3*01:01/A*01:02 and DQB1*06:03/A*01:03 (Ag^neg^/HLA^pos^ acceptor cells) restriction alleles for PTK2B and PI4K2B, respectively. After overnight coculture, T cells for PTK2B or PI4K2B were added and IFN‐γ release was measured by ELISA. Results of duplicate or triplicate wells pooled from three independent experiments represented by open, gray and black symbols are shown for T cells for PTK2B (dots; left) and PI4K2B (triangles; right).

To investigate whether indirect antigen presentation occurs for all 6 natural HLA class II ligands or for a subset of these antigens, antigen‐positive donor cells lacking the relevant HLA class II restriction alleles (Ag^pos^/HLA^neg^ donor cells) were co‐cultured with antigen‐negative acceptor cells that were positive for the HLA class II restriction alleles (Ag^neg^/HLA^pos^ acceptor cells). In our first experiments, EBV‐B cells endogenously expressing the antigens of interest were used as donor cells. As acceptor cells, HeLa cells transduced with the relevant HLA class II restriction alleles were used. After co‐culturing, T‐cell recognition was observed for 3 antigens (PTK2B, MTHFD1 and DBY), whereas donor and acceptor cells were not or hardly recognized by the respective T cells when analyzed separately (Fig. 1A, Supporting Information Figs. 1 and 2). Further analysis revealed that direct T‐cell contact was not required, since the three cytosolic proteins were efficiently transferred between cells via culture supernatants (Fig. 1B). Moreover, antigen transfer did not occur when flowthroughs of culture supernatants from 10, 30 or 100 kDa filters were loaded, indicating that intercellular transfer was mediated by particles >100 kDa.

In the experiments above, HeLa cells transduced with HLA class II were used as acceptor cells. HeLa cells are negative for all antigens analyzed except for PI4K2B. Since endogenous PI4K2B expression in HeLa complicated data interpretation for this antigen, we also developed a reversed system in which HLA class II‐negative HeLa cells transduced with wild‐type PTK2B or PI4K2B were used as donor cells and antigen‐negative EBV‐B cells endogenously expressing the relevant HLA class II alleles as acceptor cells. Using this reversed system, we confirmed that the HLA class II ligand of PTK2B can be transferred between cells, whereas no transfer was observed for the antigen of PI4K2B (Fig. 1C).

### Intercellular transfer of the HLA class II PTK2B ligand is mediated by its full‐length protein

**Figure 2:**
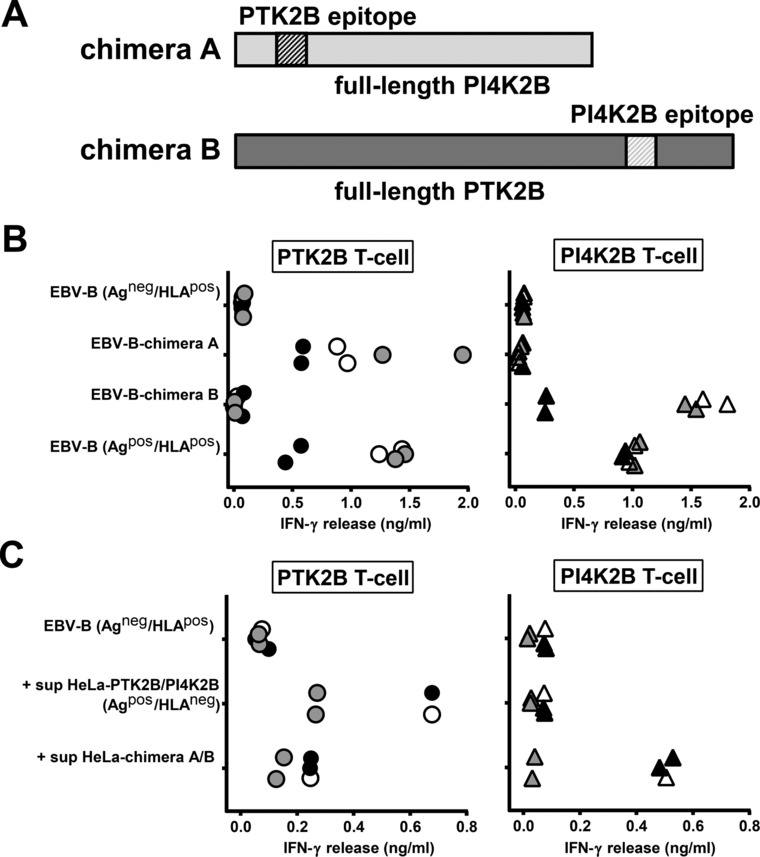
Intercellular transfer of the HLA class II ligand of PTK2B is mediated by other protein sequences than the T‐cell epitope. (A) Schematic drawing of constructs encoding full‐length PTK2B (dark gray) and PI4K2B (light gray) with exchanged T‐cell epitopes. Chimera A contains full‐length PI4K2B with the T‐cell epitope of PTK2B. Chimera B contains full‐length PTK2B with the T‐cell epitope of PI4K2B. T‐cell epitopes are indicated by hatched areas. (B) Antigen‐negative EBV‐B cells expressing HLA‐DRB3*01:01/A*01:02 and DQB1*06:03/A*01:03 (Ag^neg^/HLA^pos^ acceptor cells) were transduced with chimera A or B and tested for T‐cell recognition by IFN‐γ ELISA. Results of duplicate wells pooled from three independent experiments represented by open, gray and black symbols are shown for T cells for PTK2B (dots; left) and PI4K2B (triangles; right). (C) Antigen‐negative EBV‐B cells expressing HLA‐DRB3*01:01/A*01:02 and DQB1*06:03/A*01:03 (Ag^neg^/HLA^pos^ acceptor cells) were loaded with culture supernatants from HeLa cells transduced with wild‐type PTK2B, wild‐type PI4K2B, chimera A or chimera B. After overnight loading, T cells were added and IFN‐γ release was measured by ELISA. T cells for PTK2B (dots; left) were tested for reactivity against EBV‐B cells loaded with culture supernatants from HeLa cells transduced with wild‐type PTK2B or chimera A. T cells for PI4K2B (triangles; right) were tested for reactivity against EBV‐B cells loaded with culture supernatants from HeLa cells transduced with wild‐type PI4K2B or chimera B. Results of single or duplicate wells pooled from three independent experiments represented by open, gray and black symbols are shown.

To investigate whether indirect antigen presentation is an intrinsic property of the HLA class II ligand or whether other protein sequences are involved, we made retroviral constructs for full‐length PTK2B and PI4K2B in which the T‐cell epitopes were exchanged between both proteins. Chimera A encoded full‐length PI4K2B with the T‐cell epitope of PTK2B, whereas chimera B encoded full‐length PTK2B with the T‐cell epitope of PI4K2B (Fig. 2A). Direct presentation of the PTK2B antigen after retroviral transfer of chimera A in antigen‐negative EBV‐B cells expressing the relevant HLA class II restriction allele was in the same range as wild‐type PTK2B (Fig. 2B), confirming proper processing and presentation of the PTK2B epitope when supplied in the context of the PI4K2B protein. Direct presentation of the PI4K2B antigen after retroviral transfer of chimera B was also detected albeit with different efficiencies. To investigate indirect antigen presentation, antigen‐negative EBV‐B cells expressing the relevant HLA class II restriction alleles (acceptor cells) were loaded with culture supernatants from HLA class II‐negative HeLa cells transduced with wild‐type PTK2B, wild‐type PI4K2B, chimera A or chimera B. When supplied in their wild‐type protein context, we again demonstrated indirect presentation of PTK2B, but not for PI4K2B (Fig. 2C). However, in contrast to wild‐type PI4K2B, indirect presentation of the PI4K2B epitope was observed when supplied in the context of full‐length PTK2B (chimera B) in two out of three experiments, while indirect presentation of the PTK2B epitope in the context of full‐length PI4K2B (chimera A) was diminished as compared to wild‐type PTK2B. These data suggest that intercellular transfer of PTK2B is not dependent on the T‐cell epitope, but rather on characteristics of the full‐length protein.

**Figure 3:**
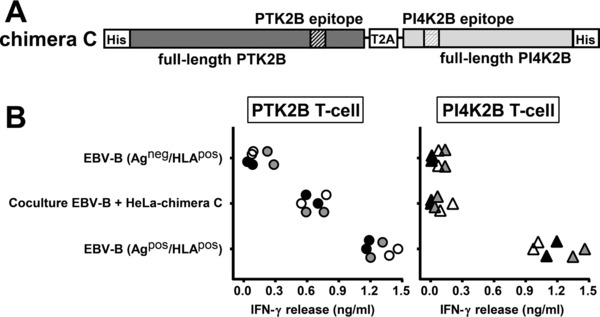
Cellular abundance of PTK2B is dependent on its protein sequence. (A) Schematic drawing of a retroviral construct encoding chimera C, which consists of full‐length PTK2B (dark gray) and PI4K2B (light gray) both fused to His‐tags and linked by a T2A sequence. T‐cell epitopes are indicated by hatched areas. (B) HeLa cells transduced with chimera C (Ag^pos^/HLA^neg^ donor cells) were cocultured with antigen‐negative EBV‐B cells expressing HLA‐DRB3*01:01/A*01:02 and DQB1*06:03/A*01:03 (Ag^neg^/HLA^pos^ acceptor cells). T‐cell recognition was measured by IFN‐γ ELISA. Results of duplicate wells pooled from three independent experiments represented by open, gray and black symbols are shown for T cells for PTK2B (dots; left) and PI4K2B (triangles; right).

To investigate the mechanism of intercellular antigen transfer in more detail, we constructed a vector encoding a chimeric protein consisting of full‐length PTK2B and PI4K2B linked by a T2A sequence (chimera C; Fig. 3A). In addition, His‐tags were fused to the N‐terminus of PTK2B and C‐terminus of PI4K2B to allow detection of both proteins by the same anti‐His antibody. T‐cell experiments again demonstrated intercellular transfer for the HLA class II ligand of PTK2B, but not for the antigen from PI4K2B (Fig. 3B). Moreover, expression of PTK2B as detected on Western blot by an anti‐His antibody was stronger than for PI4K2B (Supporting Information Fig. 3). These data indicate that cellular abundance of PTK2B is dependent on its protein sequence and that high expression of PTK2B as compared to PI4K2B cannot be explained by a difference in transcriptional activity or binding affinity of specific antibodies.

**Figure 4:**
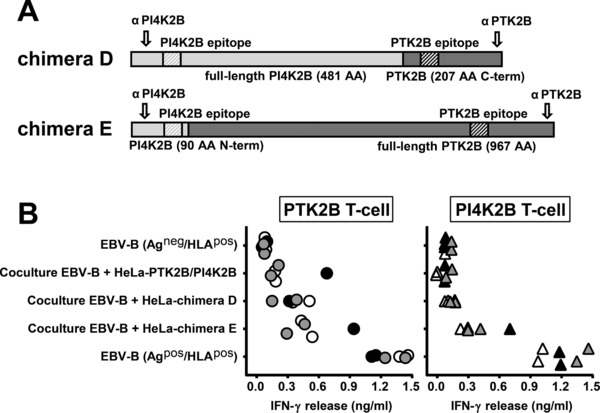
Intercellular transfer of the HLA class II ligand of PTK2B by chimeric PI4K2B‐PTK2B fusion proteins. (A) Schematic drawing of PI4K2B ‐ PTK2B fusion proteins. Chimera D consists of full‐length PI4K2B (light gray) fused to the C‐terminal 207 aa of PTK2B (dark gray), whereas chimera E consists of the N‐terminal 90 aa of PI4K2B (light gray) fused to full‐length PTK2B (dark gray). T‐cell epitopes of the respective proteins are indicated by hatched areas. Arrows indicate the binding sites of antibodies used for detection of PI4K2B and PTK2B on Western blot. (B) HLA class II‐negative HeLa cells transduced with wild‐type PTK2B, wild‐type PI4K2B, chimera D or chimera E (Ag^pos^/HLA^neg^ donor cells) were cocultured with antigen‐negative EBV‐B cells expressing HLA‐DRB3*01:01/A*01:02 and DQB1*06:03/A*01:03 (Ag^neg^/HLA^pos^ acceptor cells). T‐cell recognition was measured by IFN‐γ ELISA. Results of single or duplicate wells pooled from three independent experiments represented by open, gray and black symbols are shown for T cells for PTK2B (dots; left) and PI4K2B (triangles; right).

In an attempt to determine which protein sequences are required for intercellular transfer of the HLA class II ligand of PTK2B, two other chimeric proteins were constructed. The construct for chimera D encoded full‐length PI4K2B fused to the C‐terminal 207 aa of PTK2B and the construct for chimera E encoded the N‐terminal 90 aa of PI4K2B fused to full‐length PTK2B (Fig. 4A). Both fusion proteins contained the PI4K2B and PTK2B T‐cell epitopes as well as specific antibody epitopes at their N‐ and C‐terminal regions, respectively. T‐cell experiments demonstrated intercellular transfer of the HLA class II ligand of PTK2B upon co‐culture of HeLa cells transduced with chimera D or E with EBV‐B acceptor cells (Fig. 4B; left). T‐cell recognition of chimera D was lower than chimera E, which correlated with expression of the fusion proteins on Western blot (Supporting Information Fig. 4). Furthermore, the data for chimera E demonstrated that the HLA class II ligand of PI4K2B can be transferred between cells when fused to full‐length PTK2B (Fig. 4B; right). In conclusion, the data showed that intercellular transfer of natural HLA class II ligands that are created by genetic variants is mediated by other protein sequences than the T‐cell epitope and cannot be explained by high binding affinity of the T‐cell epitope for its HLA restriction allele or high avidity of the antigen‐specific T‐cell.

### Intercellular transfer of the HLA class II ligand of PTK2B is mediated by extracellular vesicles

**Figure 5:**
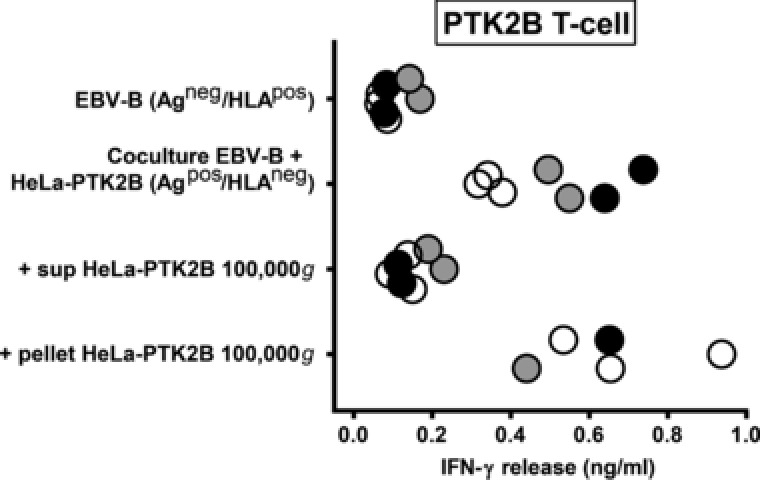
Intercellular transfer of the HLA class II ligand of PTK2B is mediated by the 100 000 × *g* pelletable fraction of cell culture conditioned medium. HLA class II‐negative HeLa cells transduced with wild‐type PTK2B (Ag^pos^/HLA^neg^ donor cells) were cocultured with antigen‐negative EBV‐B cells expressing HLA‐DRB3*01:01/A*01:02 and DQB1*06:03/A*01:03 (Ag^neg^/HLA^pos^ acceptor cells). Acceptor cells were also loaded with the 100 000 × *g* pellet and EV‐depleted supernatant of cell culture conditioned medium from HeLa cells transduced with wild‐type PTK2B. After overnight incubation, T cells were added and IFN‐γ release was measured by ELISA. Results of single, duplicate or triplicate wells pooled from three independent experiments represented by open, gray and black dots are shown for T cells for PTK2B.

To investigate whether intercellular transfer of the HLA class II ligand of PTK2B is mediated by EV, culture supernatants from HeLa cells transduced with wild‐type PTK2B were subjected to differential high speed ultracentrifugation and the 100 000 × *g* pellet was loaded on EBV‐B acceptor cells. T‐cell experiments demonstrated recognition of the 100 000 × *g* pellet, whereas the EV‐depleted supernatant collected after 100 000 × *g* centrifugation was not recognized (Fig. 5). The 100 000 × *g* pellet was confirmed to contain EV by Western blot, characterized by enrichment of tetraspanin CD9, absence of ER resident glycoprotein 96 (gp96) and presence of chaperone hsc70 (Supporting Information Fig. 5A), as well as by electron microscopy (Supporting Information Fig. 5B). As expected from T‐cell recognition data, we also detected wild‐type PTK2B in the 100 000 × *g* pellet (Supporting Information Fig. 5A). Similarly, all chimeric proteins for which intercellular transfer was observed in T‐cell experiments (chimeras B, D and E) could be detected in 100 000 × *g* pellets from culture supernatants from HeLa cells transduced with the respective chimeric genes, whereas chimera A and wild‐type PI4K2B, for which no transfer was observed, were not present (Supporting Information Fig. 4). These data showed that intercellular transfer of the HLA class II ligand of PTK2B is mediated by structures present in 100 000 × *g* pellets.

EV populations in 100 000 × *g* pellets are often contaminated with (nucleo)protein complexes. Therefore, to confirm that intercellular antigen transfer is mediated by EV, culture supernatant from HeLa cells transduced with wild‐type PTK2B was pelleted at 100 000 × *g*, labeled with the fluorescent lipid bilayer dye PKH67 and low density vesicles were separated from high density protein complexes using a density gradient 19. A total of 12 fractions with densities between 1.34 and 1.06 g/mL were collected and analyzed by high‐resolution flow cytometry 20, 21. The data indicated that PKH67 positive EV were most abundant in low density fractions 6–9 (1.21–1.07 g/mL) and showed that various EV subpopulations could be distinguished that differed in light scattering values and PKH67 staining intensity (Supporting Information Fig. 6A). Western blot analysis using antibodies against human CD9 and flotillin also indicated the enrichment of EV in fractions 7–9 (Supporting Information Fig. 6B). We next determined whether intercellular transfer of the HLA class II ligand of PTK2B was mediated by EV. The various density fractions were loaded on EBV‐B acceptor cells and T‐cell recognition was measured to assess antigen presentation. PTK2B antigen recognition, as measured by IFN‐γ release by specific T cells, was predominant in the EV containing fractions 6–7 (1.21–1.16 g/mL), confirming that intercellular transfer of the HLA class II ligand of PTK2B is mediated by EV (Supporting Information Fig. 6C). T‐cell reactivity to transferred PTK2B was not observed upon incubation with material present in fractions 2 and 9–12. Interestingly, the peak in T‐cell recognition was slightly shifted compared to the peak in number of EV, suggesting that the PTK2B antigen resides in a subpopulation of EV.

In conclusion, our data showed indirect antigen presentation for a subset of natural HLA class II ligands that are created by genetic variants and demonstrated that these antigens can be transferred between cells by full‐length proteins that are secreted in EV.

## Discussion

Priming of naive CD4 T cells by professional APC is a crucial step in the initiation of a potent immune response. The HLA class II processing pathway is perfectly equipped to efficiently present exogenous threats to CD4 T‐cell surveillance 2. However, when it comes to intracellular dangers such as viruses or malignant transformation in HLA class II‐negative cells, efficient CD4 T‐cell priming requires antigen release by cellular death or active transport of intracellular antigens to professional APC. We here demonstrate that certain cytosolic proteins can be actively transferred between viable cells and that transfer of natural HLA class II ligands can be mediated by EV. EV were isolated by a density gradient, which is essential to separate EV from contaminating non‐EV associated protein complexes 19, 22, 23. Our results confirmed previous data showing that EV harbor a distinctive set of cellular proteins and that T cells can simultaneously release EV subpopulations that differ in molecular composition and are likely formed via different biogenesis pathways 24.

Most viral infections and malignant transformations do not occur in professional APC. As such, transport of foreign or mutated cytosolic proteins from infected or transformed cells to professional APC is beneficial or even essential for efficient induction of cellular immunity. For CMV and influenza, it has been shown that viral proteins are transported from infected cells to professional APC via secreted vesicles, and that this leads to activation of CD4 T cells 25, 26. It is now well established that most cell types release EV and that these vesicles can be isolated from body fluids, such as ascites, pleural effusion, urine, breast milk and serum, emphasizing their existence in vivo 21, 27, 28, 29. Also tumor cells release EV and their downstream effects are diverse 30. Several studies show immunosuppressive properties, but there is also considerable evidence for immunosupportive effects 31, which might be explained by antigen transfer and presentation to T cells with different regulatory or effector functions.

For MHC class I restricted tumor antigens it has been reported by Wolfers et al. 22 that immunization of mice with tumor‐derived EV was more potent in tumor rejection than vaccination with tumor lysates or apoptotic bodies. They also demonstrated that differentiation antigen MART‐1/Melan‐A is sequestered as full‐length protein into EV and that this antigen can be cross presented in APC and lead to CD8 T‐cell activation. In line with these findings, we here show that PTK2B is transferred between cells as full‐length protein in EV leading to antigen presentation by HLA class II. In another mouse study in which different tumor cell vaccines were compared for their immunogenicity, targeting ovalbumin OVA to EV was shown to be superior in inducing CD8 T‐cell mediated tumor rejection as compared to membrane bound or soluble OVA 32. Increased vaccine potency upon antigen targeting to EV has also been supported by other studies 31, 33, 34, 35.

Our data demonstrate that the HLA class II ligand of PTK2B is transferred between cells by the full‐length protein in EV, but it can be argued that PTKB is not integrated in EV but adheres to the outside in a non‐specific manner. Antigen transfer by non‐specific adherence to EV, however, is unlikely, since PTK2B is an intracellular kinase and there is no evidence that it is secreted in soluble form in the extracellular space. Moreover, in our experiments, antigen transfer is measured between viable cells that rapidly grow with low percentages of cell death. There is also no evidence that PTK2B resides in the lumen of late endosomes where EV are formed. Furthermore, for EV isolation from culture supernatants, we used OptiPrep density gradient ultracentrifugation which outperforms other common isolation methods in terms of recovery and purity of EV. EV isolated by OptiPrep density gradient ultracentrifugation, for example, have been shown to lack protein(‐RNA) complexes, which contaminate EV preparations and confound functional assays 36.

In addition to PTK2B, we demonstrated indirect presentation of the HLA class II ligand of DDX3Y (DBY), which is a protein encoded by a male‐specific gene on the Y‐chromosome. It has already been shown some years ago that this protein can lead to CD4 T‐cell mediated tumor rejection independent of MHC class II expression on the tumor 12. Furthermore, recipient derived DDX3Y presented on donor APC has been reported to induce CD4 T‐cell mediated graft‐versus‐host disease in mice 37. CD4 T‐cell mediated rejection of MHC class II‐negative tumors requires several steps. First, antigens should be transferred from MHC class II‐negative tumor cells to MHC class II‐positive APC. We here showed direct evidence that DDX3Y can be transferred between cells. This is supported by studies in female mice developing a DDX3Y‐specific CD4 T‐cell response when injected with EV from male cells 38. In a next step, activated CD4 T cells need to recruit other effector cells in order to mediate rejection of MHC class II‐negative tumor cells 10, 11, 12. In mouse models macrophages and NK‐cells have been suggested to play a role, but also other immune cells might be of importance. Further studies are required to elucidate whether one special type of immune cell plays a crucial role in rejection of MHC class II‐negative tumors or whether it is mediated by an orchestrated immune response with the CD4 T‐cell as central regulator.

In conclusion, we showed indirect antigen presentation for a subset of natural HLA class II ligands and demonstrated that (neo)antigens can be transferred between cells by full‐length proteins that are secreted in EV. Indirect antigen presentation has been shown to mediate beneficial but also detrimental effects, and it is therefore relevant to take intercellular transfer into consideration when selecting (neo)antigens for cellular immunotherapy or vaccination.

## Materials and methods

### Cell culture

Cervix carcinoma HeLa and EBV‐B cell lines were cultured in IMDM (Lonza BioWhittaker, Basel, Switzerland) with 10% FCS (Cambrex, Verviers, Belgium), 1% penicillin/streptomycin (Lonza) and 1.5% L‐glutamine (Lonza). CD4 T‐cell clones were cultured in IMDM with 5% human serum, 5% FCS and 100 IU/mL IL‐2 (Chiron, Ringskiddy, Ireland), and restimulated every 10–20 days with irradiated allogeneic PBMC and 0.8 μg/mL PHA (Oxoid, Cambridge, UK) 15. For isolation of EV, HeLa cells were cultured in serum‐free IMDM with 1% penicillin/streptomycin, 1.5% L‐glutamine, 5 μg/mL insulin (Sigma‐Aldrich B.V., Zwijndrecht, The Netherlands), 5 μg/mL transferrin (Invitrogen, Breda, The Netherlands), 5 ng/mL EGF (PeproTech, Rocky Hill, NJ), 100 nM hydrocortisone (Sigma‐Aldrich B.V.) and 20 ng/mL FGF2 (Biaffin GmbH & Co KG, Kassel Niederzwehren, Germany) 39.

### Antibodies

Flow cytometry was performed on a FACS Calibur (BD Biosciences, Breda, The Netherlands) and cell sorting on a FACS Aria (BD) using PE‐labeled monoclonal antibodies against HLA‐A2 (BB7.2; BD Pharmingen, San Diego, CA), HLA‐DR (L243; BD), HLA‐DQ (1a3; Meridian Life Science, Saco, ME), NGFR (C40‐1457; BD Pharmingen) or CD20 (L27; BD). For Western blotting, mouse antibodies against hsc70 (sc‐7298; Santa Cruz Biotechnology, Santa Cruz, CA), gp96 (ab63469; Abcam, Cambridge, UK), 6xHis (ab18184; Abcam), and rabbit antibodies against CD9 (ab65230; Abcam), PTK2B (ab24798; Abcam) and PI4K2B (ab37812; Abcam) were used. As secondary antibodies, biotinylated goat anti‐mouse or anti‐rabbit antibodies (Invitrogen) were used and visualized by Streptavidin‐QDots 625 (Invitrogen) under UV illumination. For detection of EV, mouse antibodies against human CD9 (HI9a, BioLegend, Fell, Germany) and flotillin‐1 (clone 18, BD Biosciences, San Jose, CA, USA) were used.

### Retroviral constructs and transduction

HLA‐DQB1*06:03, DQB1*05:01, DQA*01:03 and DQA*01:01 were cloned in separate pLZRS vectors with truncated nerve growth factor receptor (ΔNGFR) as marker gene for DQB chains and enhanced green fluorescence protein (EGFP) for DQA chains. HLA‐DRB1*03:01, DRB1*13:01, DRB3*01:01, DRB3*02:02 and DRA*01:02 were cloned in MP71 vectors with ΔNGFR 14, 15, 18. HLA class II‐negative HeLa cells transduced with HLA class II were sorted on HLA‐DR or ‐DQ surface expression. EBV‐B cells transduced with HLA‐DRB or ‐DQB were sorted on ΔNGFR expression. The genes encoding full‐length PTK2B, MR1, PI4K2B and MTHFD1 were cloned in MP71 with human CD20 as marker gene. Full‐length DBY was cloned in pLZRS containing EGFP. Chimeric and mutated constructs were cloned by two‐step PCR. Chimera A contained full‐length PI4K2B (NM_018323) with its T‐cell epitope at 220–262 bp replaced by the PTK2B epitope. Chimera B contained full‐length PTK2B (NM_173175) with its T‐cell epitope at 2371–2410 bp replaced by the PI4K2B epitope. Chimera D contains full‐length PI4K2B fused to the last 621 bp of PTK2B. Chimera E contains the first 270 bp of PI4K2B fused to full‐length PTK2B. Chimeric constructs A, B, D and E were cloned in MP71 with CD20 as marker gene. For construct C, 6xHis‐tags were fused to the N‐terminus of PTK2B and C‐terminus of PI4K2B and both sequences were linked with a T2A sequence. The His‐PTK2B‐T2A‐PI4K2B‐His sequence was cloned in MP71 with EGFP as marker gene. All constructs were verified by DNA sequencing. Retroviral supernatants were obtained by transfecting Φnx‐A packaging cells and cells were transduced with viral supernatants in culture plates coated with recombinant human fibronectin CH 296 (Takara Shuzo, Otsu, Shiga, Japan) 40.

### Antigen presentation assays

Stimulator cells (10 000 or 30 000 cells/well) were co‐incubated with CD4 T cells (5000 cells/well) overnight at 37°C in 96‐well plates. As CD4 T cells, clones for PI4K2B 14, PTK2B 15, MTHFD1 15, LY75 15, MR1 15 and DBY 18 were used. In intercellular transfer experiments, donor and acceptor cells were plated at 1:1 ratios in 96‐wells plates overnight before adding the T cells. As acceptor cells, HeLa cells transduced with LZRS encoding the invariant chain and human CD80 as marker gene was used. HeLa‐Ii cells have been demonstrated to efficiently process and present HLA class II ligands 41. For loading with recombinant proteins, *E.coli* with a pKE‐1 vector encoding the respective antigen under an IPTG‐inducible promoter were grown to OD_600_ of 0.5 with 50 μg/mL ampicillin (Sigma‐Aldrich B.V.) and protein expression was induced by 1 mM IPTG for 4 h (Promega, Madison, WI). Subsequently, bacteria were opsonized by adding human serum with 17% (vol/vol) complement (Sigma‐Aldrich B.V.) for 1 h. Target cells were pulsed with complement‐opsonized bacteria in IMDM with 10% FCS and 30 μg/mL gentamycin (Sigma‐Aldrich B.V.) overnight at 37°C before T cells were added. Culture supernatants were depleted of cells by centrifugation with 300 × *g* for 10 min and separated through 5, 30 (Vivaproducts, Littleton, MA) or 100 (Millipore, Amsterdam, The Netherlands) kDa filters. Concentrated flow through fractions (50 μL) or isolated 100 000 × *g* fractions (50 μL) were loaded on target cells in 96‐well plates overnight before T cells were added. Cytokine release was measured in 50 μL supernatants by IFN‐γ ELISA (Sanquin, Burton upon Trent, UK).

### Western blot analysis

Cells were harvested, washed with PBS and resuspended in 1% Triton‐X (Sigma‐Aldrich B.V.) lysis buffer containing protease inhibitors (Roche, Woerden, The Netherlands) for 20 min on ice. Whole cell lysates were obtained after centrifugation at 10 000 × *g* for 30 min. Protein concentration was assessed by the Bradford assay (Bio‐Rad Laboratories B.V., Veenendaal, The Netherlands). SDS‐Page was run with 10–25 μg protein in each lane on precast NuPage^®^ Novex 10% Bis‐Tris Mini gels (Invitrogen) for 35 min at 30V under denaturing conditions. Gels were blotted on PVDF membranes using XCell SureLock^®^ Mini‐Cell blotting system (Invitrogen), blocked overnight with 5% BSA in PBS with 0.05% Tween‐20 and subsequently incubated with primary antibody, biotinylated secondary antibody and streptavidin‐QDots_ 625_. Each incubation step was performed for 1 h at RT. Antibody binding was visualized under UV illumination.

### Isolation of extracellular vesicles

HeLa cells were grown in serum‐free medium for 3 days. Culture supernatant was depleted of cells by centrifugation at 300 × *g* for 10 min. Supernatants were subsequently depleted of cellular debris by centrifugation at 3000 × *g* for 20 min, followed by centrifugation at 10 000 × *g* for 30 min. Finally, supernatants were centrifuged at 100 000 × *g* for 15 h, pellets were resuspended in PBS and centrifuged again at 100 000 × *g* for 15 h in a Beckman‐Coulter SW28 rotor. Pellets collected at 100 000 × *g* were resuspended in 150–200 μL culture medium for T‐cell experiments, 50 μL 1% Triton‐X lysis buffer for Western blot analysis or PBS/4% glutaraldehyde (Brunschwig Chemie, Amsterdam, The Netherlands) for analysis by transmission electron microscopy. For isolation of EV by density gradient ultracentrifugation, supernatants were centrifuged at 100 000 × *g* for 1.05 h, pellets were washed in PBS and centrifuged again at 100 000 × *g* for 1.05 h in a SW28 rotor. Pelleted particles were stained with PKH67 (Sigma Aldrich, Zwijndrecht, The Netherlands) as described previously 20, 21. Pellets were transferred to SW40 tubes and mixed with 1.5 mL 60% OptiPrep (Progen, Heidelberg, Germany) and overlayed with 15 additional OptiPrep fractions ranging from 50 to 10% in increments of 700 μL. Density gradients were centrifuged for 16 h at 192 000 × *g* in a SW40Ti rotor in a Beckman Coulter Optima L‐90K ultracentrifuge. A total of 12 fractions (1.34‐1.06 g/mL) were collected. Each fraction was diluted 10‐fold and loaded on EBV‐B cells. After overnight incubation, EBV‐B cells were washed and antigen recognition by CD4 T cells was measured by IFN‐γ ELISA. The BD Influx^TM^ flow cytometer (Becton Dickinson, Breda, The Netherlands) with optimized configuration was used for high resolution flow cytometric analysis of vesicles in different density fractions as described previously 20. The system was triggered on PKH67 fluorescence signals derived from the EV and thresholding on this fluorescence channel allowed discrimination between EV and noise events.

## Conflict of interest

The authors declare no commercial or financial conflict of interest.

**Abbreviations**
APC: antigen presenting cells
EV: extracellular vesicles

## Supporting information

Figure 1. Intercellular transfer of the natural HLA class II ligand of PTK2B.
Figure 2. Intercellular transfer of natural HLA class II ligands that are created by genetic variants.
Figure 3. Cellular abundance of PTK2B is dependent on its protein sequence.
Figure 4. Chimeric PTK2B‐PI4K2B proteins in 100,000*g* fractions.
Figure 5. Analysis of the 100,000*g* fraction from HeLa cells transduced with wild‐type PTK2B.
Figure 6. Intercellular transfer of the HLA class II ligand of PTK2B is mediated by extracellular vesicles.

Peer review correspondence

## References

[1] Toes, R. E. , Schoenberger, S. P. , van der Voort, E. I. , Offringa, R. and Melief, C. J. , CD40‐CD40 ligand interactions and their role in cytotoxic T lymphocyte priming and anti‐tumor immunity. Semin. Immunol. 1998 10: 443–448. 9826577 10.1006/smim.1998.0147

[2] Vyas, J. M. , Van der Veen, A. G. and Ploegh, H. L. , The known unknowns of antigen processing and presentation. Nat. Rev. Immunol. 2008 8: 607–618. 18641646 10.1038/nri2368 PMC2735460

[3] Janssen, E. M. , Lemmens, E. E. , Wolfe, T. , Christen, U. , von Herrath, M. G. and Schoenberger, S. P. , CD4+ T cells are required for secondary expansion and memory in CD8+ T lymphocytes. Nature. 2003 421: 852–856. 12594515 10.1038/nature01441

[4] Rocha, P. N. , Plumb, T. J. , Crowley, S. D. and Coffman, T. M. , Effector mechanisms in transplant rejection. Immunol. Rev. 2003 196: 51–64. 14617197 10.1046/j.1600-065x.2003.00090.x

[5] Black, C. A. , Delayed type hypersensitivity: current theories with an historic perspective. Dermatol Online J. 1999 5: 7. 10673450

[6] Gokmen, M. R. , Lombardi, G. and Lechler, R. I. , The importance of the indirect pathway of allorecognition in clinical transplantation. Curr. Opin. Immunol. 2008 20: 568–574. 18655831 10.1016/j.coi.2008.06.009

[7] Libby, P. and Pober, J. S. , Chronic rejection. Immunity 2001 14: 387–397. 11336684 10.1016/s1074-7613(01)00119-4

[8] Anderson, B. E. , McNiff, J. M. , Jain, D. , Blazar, B. R. , Shlomchik, W. D. and Shlomchik, M. J. , Distinct roles for donor‐ and host‐derived antigen‐presenting cells and costimulatory molecules in murine chronic graft‐versus‐host disease: requirements depend on target organ. Blood 2005 105: 2227–2234. 15522961 10.1182/blood-2004-08-3032

[9] Brand, O. , Gough, S. and Heward, J. , HLA, CTLA‐4 and PTPN22: the shared genetic master‐key to autoimmunity? Expert Rev Mol Med 2005 7: 1–15. 10.1017/S1462399405009981 16229750

[10] Corthay, A. , Skovseth, D. K. , Lundin, K. U. , Rosjo, E. , Omholt, H. , Hofgaard, P. O. , Haraldsen, G. et al., Primary antitumor immune response mediated by CD4+ T cells. Immunity 2005 22: 371–383. 15780993 10.1016/j.immuni.2005.02.003

[11] Mumberg, D. , Monach, P. A. , Wanderling, S. , Philip, M. , Toledano, A. Y. , Schreiber, R. D. and Schreiber, H. , CD4(+) T cells eliminate MHC class II‐negative cancer cells in vivo by indirect effects of IFN‐gamma. Proc. Natl. Acad. Sci. USA 1999 96: 8633–8638. 10411927 10.1073/pnas.96.15.8633 PMC17568

[12] Perez‐Diez, A. , Joncker, N. T. , Choi, K. , Chan, W. F. , Anderson, C. C. , Lantz, O. and Matzinger, P. , CD4 cells can be more efficient at tumor rejection than CD8 cells. Blood 2007 109: 5346–5354. 17327412 10.1182/blood-2006-10-051318 PMC1890845

[13] Griffioen, M. , Van Bergen, C. A. M. and Falkenburg, J. H. F. , Autosomal minor histocompatibility antigens; how genetic variants create diversity in immune targets. Front Immunol. 2016 2016: 7–100. 10.3389/fimmu.2016.00100 PMC4791598 27014279

[14] Griffioen, M. , van der Meijden, E. D. , Slager, E. H. , Honders, M. W. , Rutten, C. E. , van Luxemburg‐Heijs, S. A. P. , et al., Identification of phosphatidylinositol 4‐kinase type II beta as HLA class II‐restricted target in graft versus leukemia reactivity. Proc. Natl. Acad. Sci. USA 2008 105: 3837–3842. 18316730 10.1073/pnas.0712250105 PMC2268788

[15] Stumpf, A. N. , van der Meijden, E. D. , van Bergen, C. A. M. , Willemze, R. , Falkenburg, J. H. F. and Griffioen, M. , Identification of 4 new HLA‐DR‐restricted minor histocompatibility antigens as hematopoietic targets in antitumor immunity. Blood 2009 114: 3684–3692. 19706888 10.1182/blood-2009-03-208017

[16] Schumacher, T. N. and Schreiber, R. D. , Neoantigens in cancer immunotherapy. Science 2015 348: 69–74. 25838375 10.1126/science.aaa4971

[17] Tran, E. , Robbins, P. F. and Rosenberg, S. A. , ‘Final common pathway’ of human cancer immunotherapy: targeting random somatic mutations. Nat. Immunol. 2017 18: 255–262. 28198830 10.1038/ni.3682 PMC6295671

[18] Vogt, M. H. , van den Muijsenberg, J. W. , Goulmy, E. , Spierings, E. , Kluck, P. , Kester, M. G. , van Soest, R. A. , et al., The DBY gene codes for an HLA‐DQ5‐restricted human male‐specific minor histocompatibility antigen involved in graft‐versus‐host disease. Blood 2002 99: 3027–3032. 11929796 10.1182/blood.v99.8.3027

[19] Lotvall, J. , Hill, A. F. , Hochberg, F. , Buzas, E. I. , Di Vizio, D. , Gardiner, C. , Gho, Y. S. , et al., Minimal experimental requirements for definition of extracellular vesicles and their functions: a position statement from the International Society for Extracellular Vesicles. J. Extracell Vesicles 2014 3: 26913. 25536934 10.3402/jev.v3.26913 PMC4275645

[20] van der Vlist, E. J. , Nolte‐’t Hoen, E. N. , Stoorvogel, W. , Arkesteijn, G. J. and Wauben, M. H. , Fluorescent labeling of nano‐sized vesicles released by cells and subsequent quantitative and qualitative analysis by high‐resolution flow cytometry. Nat. Protoc. 2012 7: 1311–1326. 22722367 10.1038/nprot.2012.065

[21] Nolte‐’t Hoen, E. N. , van der Vlist, E. J. , Aalberts, M. , Mertens, H. C. , Bosch, B. J. , Bartelink, W. , Mastrobattista, E. , et al., Quantitative and qualitative flow cytometric analysis of nanosized cell‐derived membrane vesicles. Nanomedicine 2012 8: 712–720. 22024193 10.1016/j.nano.2011.09.006 PMC7106164

[22] Wolfers, J. , Lozier, A. , Raposo, G. , Regnault, A. , Thery, C. , Masurier, C. , Flament, C. , et al., Tumor‐derived exosomes are a source of shared tumor rejection antigens for CTL cross‐priming. Nat. Med. 2001 7: 297–303. 11231627 10.1038/85438

[23] Sahu, R. , Kaushik, S. , Clement, C. C. , Cannizzo, E. S. , Scharf, B. , Follenzi, A. , Potolicchio, I. , et al., Microautophagy of cytosolic proteins by late endosomes. Dev. Cell. 2011 20: 131–139. 21238931 10.1016/j.devcel.2010.12.003 PMC3025279

[24] Kowal, J. , Arras, G. , Colombo, M. , Jouve, M. , Morath, J. P. , Primdal‐Bengtson, B. , Dingli, F. , et al., Proteomic comparison defines novel markers to characterize heterogeneous populations of extracellular vesicle subtypes. Proc. Natl. Acad. Sci. USA 2016 113: E968–E977. 26858453 10.1073/pnas.1521230113 PMC4776515

[25] Testa, J. S. , Apcher, G. S. , Comber, J. D. and Eisenlohr, L. C. , Exosome‐driven antigen transfer for MHC class II presentation facilitated by the receptor binding activity of influenza hemagglutinin. J. Immunol. 2010 185: 6608–6616. 21048109 10.4049/jimmunol.1001768 PMC3673890

[26] Walker, J. D. , Maier, C. L. and Pober, J. S. , Cytomegalovirus‐infected human endothelial cells can stimulate allogeneic CD4+ memory T cells by releasing antigenic exosomes. J. Immunol. 2009 182: 1548–1559. 19155503 10.4049/jimmunol.182.3.1548 PMC2630120

[27] Kharaziha, P. , Ceder, S. , Li, Q. and Panaretakis, T. , Tumor cell‐derived exosomes: a message in a bottle. Biochim Biophys Acta. 2012 1826: 103–111. 22503823 10.1016/j.bbcan.2012.03.006

[28] Keller, S. , Sanderson, M. P. , Stoeck, A. and Altevogt, P. , Exosomes: from biogenesis and secretion to biological function. Immunol. Lett. 2006 107: 102–108. 17067686 10.1016/j.imlet.2006.09.005

[29] Keller, S. , Ridinger, J. , Rupp, A. K. , Janssen, J. W. and Altevogt, P. , Body fluid derived exosomes as a novel template for clinical diagnostics. J. Transl. Med. 2011 9: 86. 21651777 10.1186/1479-5876-9-86 PMC3118335

[30] Thery, C. , Ostrowski, M. and Segura, E. , Membrane vesicles as conveyors of immune responses. Nat. Rev. Immunol. 2009 9: 581–593. 19498381 10.1038/nri2567

[31] Naslund, T. I. , Gehrmann, U. , Qazi, K. R. , Karlsson, M. C. and Gabrielsson, S. , Dendritic cell‐derived exosomes need to activate both T‐ and B‐cells to induce antitumor immunity. J. Immunol. 2013 190: 2712–2719. 23418627 10.4049/jimmunol.1203082

[32] Zeelenberg, I. S. , Ostrowski, M. , Krumeich, S. , Bobrie, A. , Jancic, C. , Boissonnas, A. , Delcayre, A. , et al., Targeting tumor antigens to secreted membrane vesicles in vivo induces efficient antitumor immune responses. Cancer Res. 2008 68: 1228–1235. 18281500 10.1158/0008-5472.CAN-07-3163

[33] Rountree, R. B. , Mandl, S. J. , Nachtwey, J. M. , Dalpozzo, K. , Do, L. , Lombardo, J. R. , Schoonmaker, P. L. , et al., Exosome targeting of tumor antigens expressed by cancer vaccines can improve antigen immunogenicity and therapeutic efficacy. Cancer Res. 2011 71: 5235–5244. 21670078 10.1158/0008-5472.CAN-10-4076

[34] Hartman, Z. C. , Wei, J. , Glass, O. K. , Guo, H. , Lei, G. , Yang, X. Y. , Osada, T. , et al., Increasing vaccine potency through exosome antigen targeting. Vaccine 2011 29: 9361–9367. 22001882 10.1016/j.vaccine.2011.09.133 PMC3350974

[35] Gehrmann, U. , Hiltbrunner, S. , Georgoudaki, A. M. , Karlsson, M. C. , Naslund, T. I. and Gabrielsson, S. , Synergistic induction of adaptive antitumor immunity by codelivery of antigen with alpha‐galactosylceramide on exosomes. Cancer Res. 2013 73: 3865–3876. 23658368 10.1158/0008-5472.CAN-12-3918

[36] Van Deun, J. , Mestdagh, P. , Sormunen, R. , Cocquyt, V. , Vermaelen, K. , Vandesompele, J. , Bracke, M. , et al., The impact of disparate isolation methods for extracellular vesicles on downstream RNA profiling. J. Extracell. Vesicles 2014 3: 1–14. 10.3402/jev.v3.24858 PMC4169610 25317274

[37] Wang, X. , Li, H. , Matte‐Martone, C. , Cui, W. , Li, N. , Tan, H. S. , Roopenian, D. et al., Mechanisms of antigen presentation to T cells in murine graft‐versus‐host disease: cross‐presentation and the appearance of cross‐presentation. Blood 2011 118: 6426–6437. 21963602 10.1182/blood-2011-06-358747 PMC3236124

[38] Thery, C. , Duban, L. , Segura, E. , Veron, P. , Lantz, O. and Amigorena, S. , Indirect activation of naive CD4+ T cells by dendritic cell‐derived exosomes. Nat. Immunol. 2002 3: 1156–1162. 12426563 10.1038/ni854

[39] Hutchings, S. E. and Sato, G. H. , Growth and maintenance of HeLa cells in serum‐free medium supplemented with hormones. Proc. Natl. Acad. Sci. USA 1978 75: 901–904. 273251 10.1073/pnas.75.2.901 PMC411365

[40] Heemskerk, M. H. M. , Hoogeboom, M. , de Paus, R. A. , Kester, M. G. D. , van der Hoorn, M. A. W. G. , Goulmy, E. , Willemze, R. et al., Redirection of antileukemic reactivity of peripheral T‐lymphocytes using gene transfer of minor histocompatibility antigen HA‐2‐specific T‐cell receptor complexes expressing a conserved alpha joining region. Blood 2003 102: 3530–3540. 12869497 10.1182/blood-2003-05-1524

[41] Kremer, A. N. , van der Meijden, E. D. , Honders, M. W. , Goeman, J. J. , Wiertz, E. J. , Falkenburg, J. H. F. and Griffioen, M. , Endogenous HLA class II epitopes that are immunogenic in vivo show distinct behavior toward HLA‐DM and its natural inhibitor HLA‐DO. Blood 2012 120: 3246–3255. 22889757 10.1182/blood-2011-12-399311

