# Supplementary material for: Natural T‐cell ligands that are created by genetic variants can be transferred between cells by extracellular vesicles
Source: Eur J Immunol. 2018 Aug 17;48(10):1621–31. doi: 10.1002/eji.201747152 (PMC6220790; doi:10.1002/eji.201747152)
Supplement: Supplementary file 1 — Figure 1. Intercellular transfer of the natural HLA class II ligand of PTK2B. Figure 2. Intercellular transfer of natural HLA class II ligands that are created by genetic variants. Figure 3. Cellular abundance of PTK2B is dependent on its protein sequence. Figure 4. Chimeric PTK2B‐PI4K2B proteins in 100,000g fractions. Figure 5. Analysis of the 100,000g fraction from HeLa cells transduced with wild‐type PTK2B. Figure 6. Intercellular transfer of the HLA class II ligand of PTK2B is mediated by extracellular vesicles. [file EJI-48-1621-s001.pdf]

European Journal of Immunology

Supporting Information
for
DOI 10.1002/eji.201747152

Anita N. Kremer, Marijke I. Zonneveld, Andreas E. Kremer, Edith D. van der Meijden, J.H. Frederik Falkenburg, Marca H.M. Wauben, Esther N.M. Nolte-‘t Hoen and Marieke Griffioen

Natural T-cell ligands that are created by genetic variants can be transferred between cells by extracellular vesicles

© 2018 The Authors. *European Journal of Immunology* published by WILEY-VCH Verlag GmbH & Co. KGaA, Weinheim.

Supporting Information

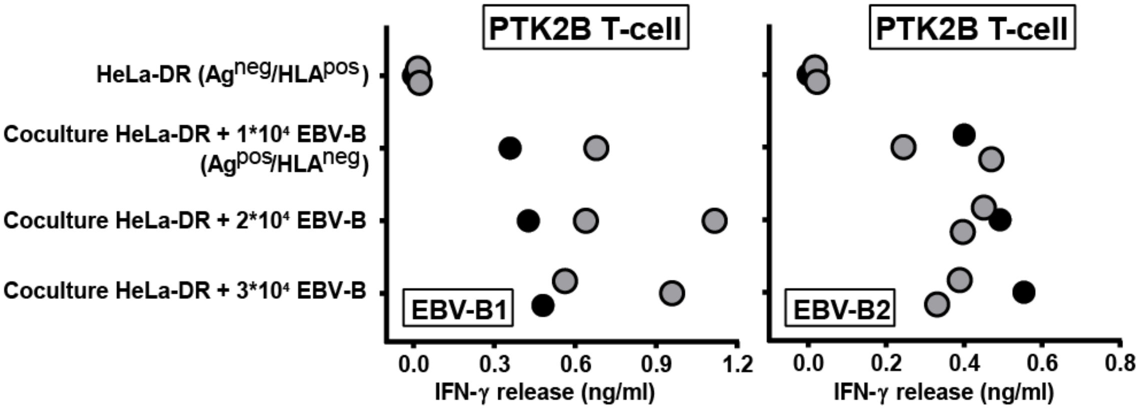

Supplemental Figure 1. Intercellular transfer of the natural HLA class II ligand of PTK2B. HeLa acceptor cells transduced with HLA-DRB3*01:01/A*01:02 were cocultured with variable numbers of antigen-positive EBV-B1 (left) or EBV-B2 (right) donor cells lacking the relevant HLA-DRB3*01:01/A*01:02 restriction allele. Results of single or duplicate wells from two independent experiments represented by black and grey dots are shown.

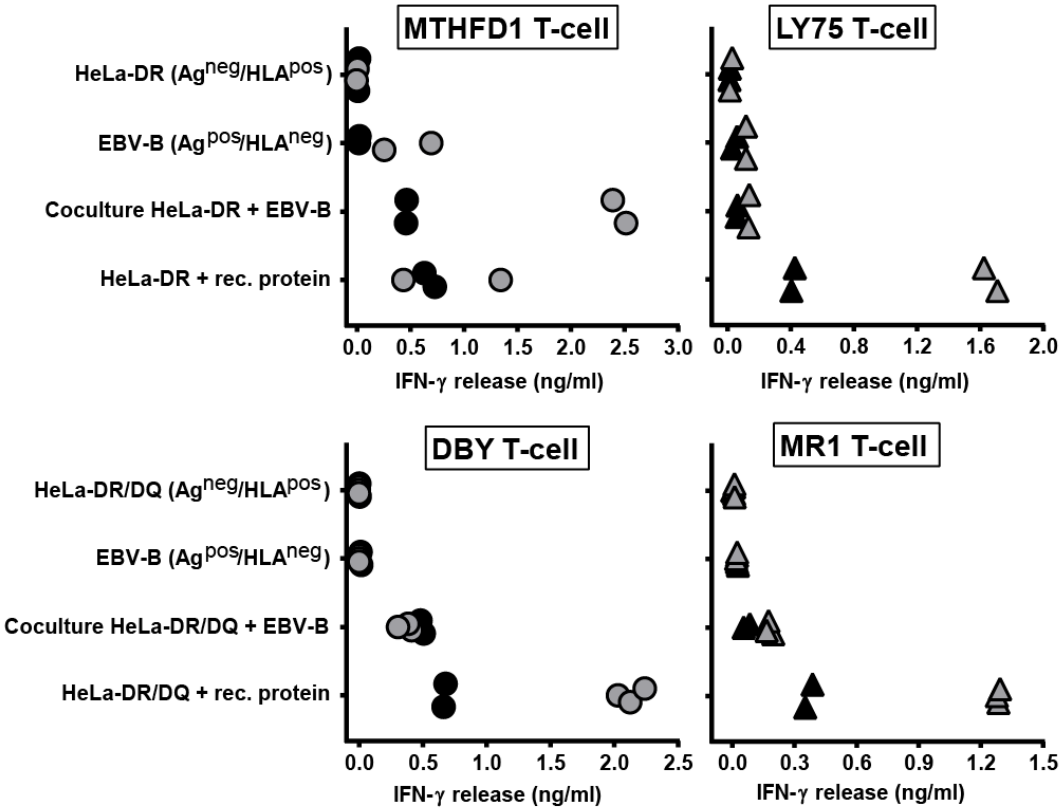

Supplemental Figure 2. Intercellular transfer of natural HLA class II ligands that are created by genetic variants. Antigen-positive EBV-B cells lacking the relevant HLA class II restriction alleles (Ag^{pos}/HLA^{neg} donor cells) were co-cultured with antigen-negative HeLa cells retrovirally transduced with HLA-DRB1*03:01/A*01:02, DRB1*13:01/A*01:02, DQB1*05:01/A*01:01 or DRB3*02:02/A*01:02 (Ag^{neg}/HLA^{pos} acceptor cells, which are the HLA class II restriction alleles for MTHFD1, LY75, DBY and MR1, respectively. As positive control, HLA class II-transduced HeLa cells were exogenously loaded with recombinant MTHFD1, DBY, LY75 or MR1 proteins. Antigen processing and presentation into HLA class II was assessed by measuring T-cell recognition in IFN-γ ELISA. Results of duplicate or triplicate wells from two independent experiments represented by grey and black symbols are shown for T-cells for MTHFD1 (dots; upper left), LY75 (triangles, upper right), DBY (dots; lower left) and MR1 (triangles; lower right) are shown.

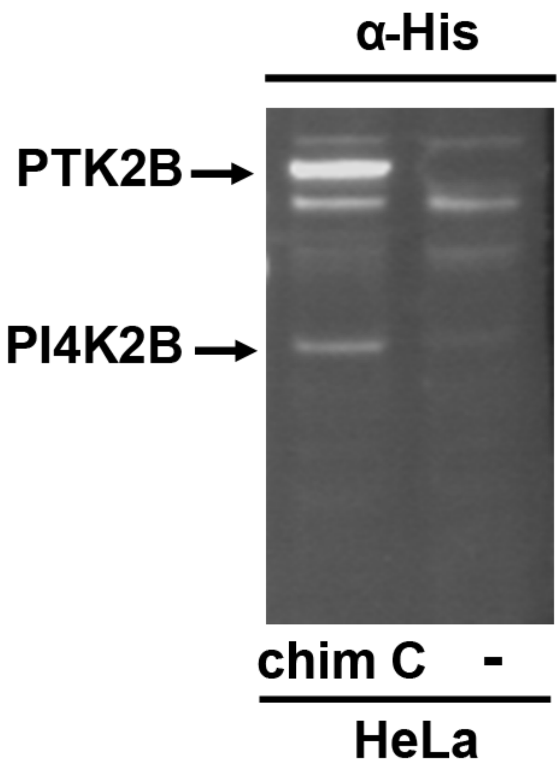

Supplemental Figure 3. Cellular abundance of PTK2B is dependent on its protein sequence. Expression of PTK2B and PI4K2B in HeLa cells transduced with chimera C was analyzed on Western blot using an α-His antibody. Results are from a single experiment. Despite equimolar translation of PTK2B and PI4K2B by a single retroviral transcript, strong expression of PTK2B was observed, while expression of PI4K2B was weak.

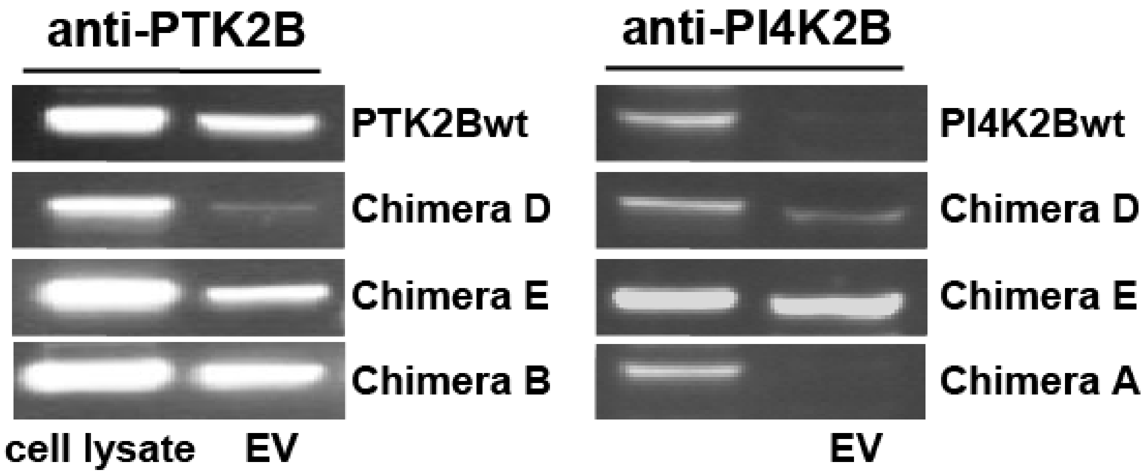

Supplemental Figure 4. Chimeric PTK2B-PI4K2B proteins in 100,000*g* fractions. Western blot with whole cell lysates and 100,000*g* pelletable fractions from cell culture conditioned medium from HeLa cells transduced with wild-type PTK2B, wild-type PI4K2B or chimeric PI4K2B-PTK2B proteins (chimera A, B, D and E). The anti-PI4K2B antibody is specific for an epitope at the N-terminus of PI4K2B, while the anti-PTK2B antibody recognizes an epitope at the C-terminus of PTK2B. Results are from a single experiment.

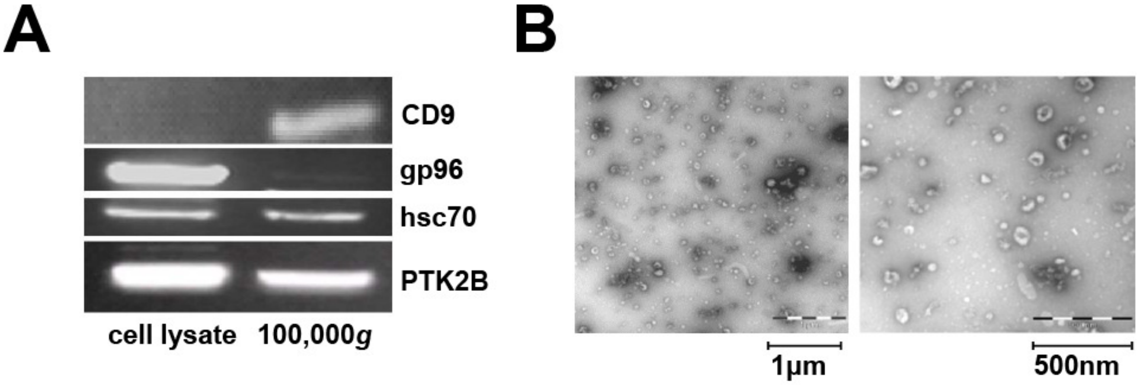

Supplemental Figure 5. Analysis of the 100,000*g* fraction from HeLa cells transduced with wild-type PTK2B.

(A) Whole cell lysate and lysate from the 100,000*g* pelletable fraction from cell culture conditioned medium from HeLa cells transduced with wild type PTK2B were analyzed for the presence of CD9, gp96, hsc70 as well as PTK2B on Western blot. Results are from a single experiment.

(B) 100,000*g* pellets were analyzed by electron microscopy. Visualization of vesicles with sizes of 30-100 nm confirmed the presence of EV. Results are from a single experiment.

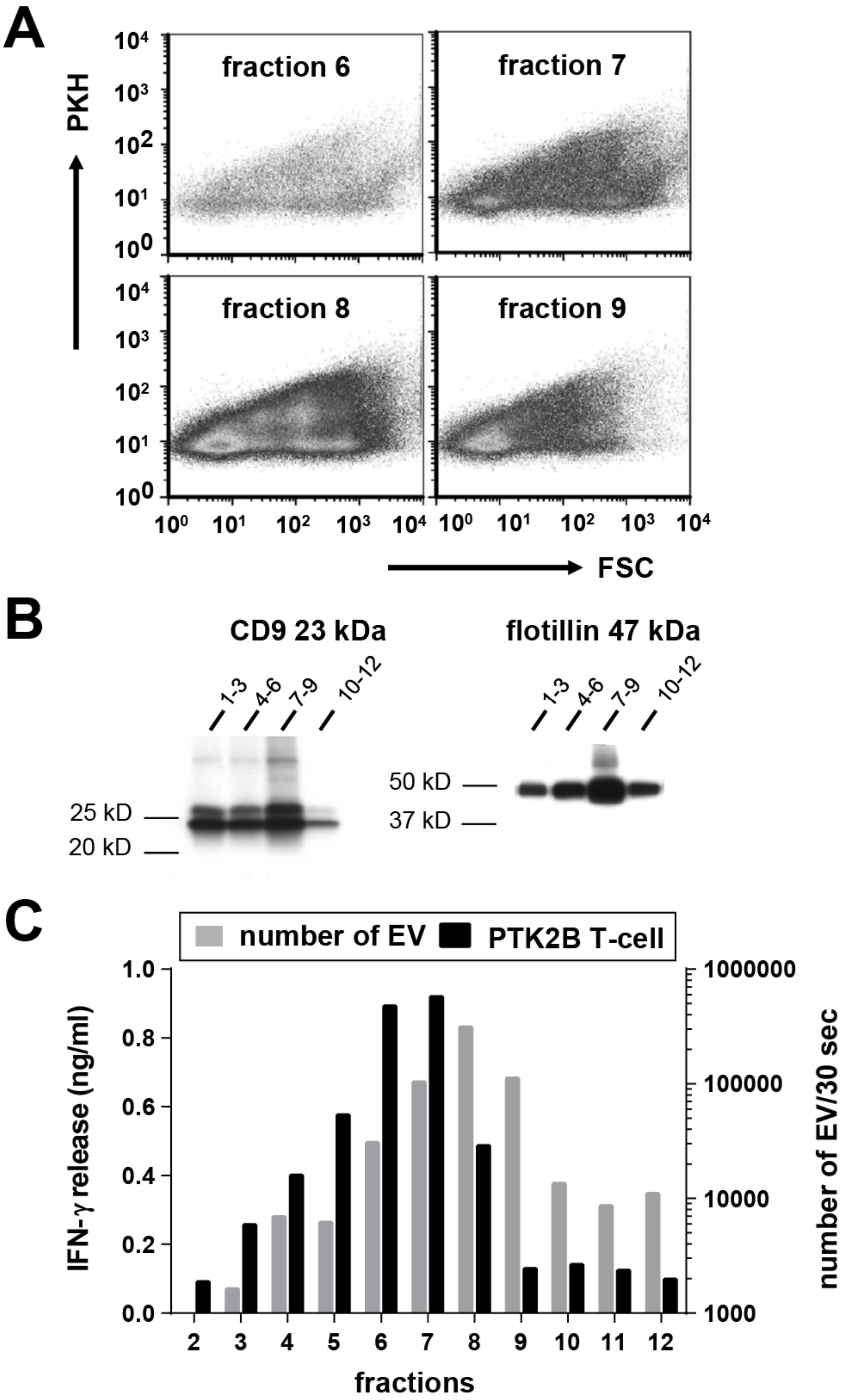
A
fraction 6
fraction 7
fraction 8
fraction 9
PKH
FSC
B
CD9 23 kDa
flotillin 47 kDa
1-3
4-6
7-9
10-12
25 kD
20 kD
50 kD
37 kD
C
number of EV
PTK2B T-cell
IFN-γ release (ng/ml)
number of EV/30 sec
fractions

Supplemental Figure 6. Intercellular transfer of the HLA class II ligand of PTK2B is mediated by extracellular vesicles.

(A) The 100,000*g* pellet collected from culture supernatant from HLA class II-negative HeLa cells transduced with wild-type PTK2B was stained with PKH67 and EV were separated from protein aggregates by density gradient ultracentrifugation. A total of 12 different fractions with densities between 1.34-1.06 g/ml were collected and analyzed by high resolution flow cytometry. Indicated are PKH67 fluorescence (Y-axis) and reduced wide-angle (rw)-FSC (X-axis) for fraction 6 (1.21 g/ml), 7 (1.16 g/ml), 8 (1.12 g/ml) and 9 (1.07 g/ml). Results are from a single experiment.

(B) Pools of three consecutive density fractions were analyzed for the presence of CD9 (23 kDa) and flotillin (47 kDa) on Western blot. Results are from a single experiment.

(C) The different density fractions were loaded on antigen-negative EBV-B acceptor cells expressing HLA-DRB3*01:01/A*01:02. After overnight incubation, T-cell recognition was measured by IFN-γ ELISA in a single experiment. Indicated are the number of EV per 30 sec in the indicated density gradient fractions as determined by high resolution flow cytometry (right Y-axis; grey bars) and release of IFN-γ (ng/ml) by T-cells for PTK2B (left Y-axis; black bars) in response to these fractions in single wells.
