# Supplementary material for: Natural T‐cell ligands that are created by genetic variants can be transferred between cells by extracellular vesicles
Source: Eur J Immunol. 2018 Aug 17;48(10):1621–31. doi: 10.1002/eji.201747152 (PMC6220790; doi:10.1002/eji.201747152)
Supplement: Supplementary file 2 — Peer review correspondence [file EJI-48-1621-s002.pdf]

Reply to the reviewers

Reviewer: 1

1. The revised article by Kremer et al maybe answered my previous comments, but the reply to reviewer is presented in such a confusing way (reply to all comments as a block, instead of each reply following each comment !), the novel figures, now displaying dots for individual biological replicates as requested, are so confusing, and the novel text presenting them is so inaccurate (first novel paragraph indicating that figures 1A-C and suppl 1 are all performed with B-EBV as both donor and acceptor, which is not the case), that I am not able to reevaluate their work.

<u>Reply</u>

We included and re-structured our previous reply to the reviewers (see below), made new figures showing dots for individual biological replicates as requested but in separate panels for different T cell clones. We also checked and corrected the text and excluded the figure in which B-EBV were used as both donor and acceptor to prevent confusion.

2. In addition, they are still displaying most data as supplementary figure, even those that are really important to evaluate validity of their experiments.

<u>Reply</u>

On request of the reviewer, we included all supplemental figures in the revised manuscript.

3. The authors, must re-do the figures again. I cannot figure out why they have now mixed in each panel results obtained using 2 different T cell clones, resulting in symbols on top of each other: it is really impossible to understand these figures. Please, use the same design as in the previous version (one panel for each T cell clone), but showing dots for values obtained for each of the 2 wells of each of the 2 experiments (as done in the present figures).

<u>Reply</u>

We made new figures according to reviewer’s suggestions showing dots for individual biological replicates but in separate panels for different T cell clones.

4. In addition, the text does not describe accurately the results: for instance the very ellyptic way of presenting figures 1A-C and suppl 1 does not reflect the experimental situation: in suppl 1A the acceptor cells are Hela, and not EBV as suggested by the text.

Reply

Indeed, the acceptor cells in supplemental Figure 1A are HeLa cells and not EBV-B cells as suggested in the text. We changed the text and excluded the figure in which B-EBV were used as both donor and acceptor to prevent confusion.

5. Given that EJI does not impose strong length restrictions, and given that many experiments that give important information (like the nature of Ag that can actually be transferred: fig1), are still displayed in suppl figures, this article is very difficult to follow and its value difficult to appreciate.

Reply

On request of the reviewer, we included all supplemental figures in the revised manuscript.

Reviewer: 2

Thank you for carefully and thoughtfully addressing the reviwers' comments. The explanations and additional experiments have significantly imptoved the impact of this manuscript.

Previous reply to the reviewers

Reviewer 1

1. When looking at the right panel of figure 1A, some presentation of the PI4K2B antigen is observed in this co-culture setting. The authors interpret it, however, as no transfer of this Ag. Indeed, when culture supernatant is used (Fig1B), no presentation is observed. The authors should at least discuss this observation when they describe the results.

Reply

There is indeed some presentation of PI4K2B in Figure 1A (right) when antigen-positive EBV-B cells lacking the relevant HLA class II allele (donor cells) were cocultured with HeLa cells transduced with the relevant HLA class II restriction allele (acceptor cells). HeLa cells are negative for all antigens of interest except for PI4K2B. Since endogenous PI4K2B expression by HeLa cells complicated data interpretation for this antigen, we also developed a reversed system in which HLA class II-negative HeLa cells transduced with wild-type PI4K2B or PTK2B (donor cells) were cocultured with antigen-negative EBV-B cells expressing the relevant HLA class II allele (acceptor cells) (Figure 1D of revised manuscript). In this reversed system, no intercellular transfer of PI4K2B is observed. Intercellular transfer of PI4K2B was also not observed in coculture experiments using antigen-positive EBV-B cells lacking the relevant HLA class II restriction alleles as donor cells and antigen-negative EBV-B cells that are positive for the relevant HLA class II restriction alleles as acceptor cells (data not shown). In the revised manuscript, we clearly describe that HeLa cells are negative for all antigens of interest except for the PI4K2B antigen and that this is the reason why we developed the reversed system as shown in Figure 1D (page 7 of revised manuscript).

2. Can the authors explain how the respective amounts of antigen- and HLA-expressing cells were chosen for the coculture and if they had performed a dose-response experiment?

Reply

We performed a dose-response experiment as included in Figure 1B of the revised manuscript. Based on these data, we decided to coculture 10,000-30,000 donor cells and 10,000-30,000 acceptor cells in a 1:1 ratio as described in “Antigen presentation assays” in the Materials and Methods of the revised manuscript.

3. In Figure 2 : the authors express chimeric proteins including mixed sequences of the transferred and not transferred antigen, and conclude that insertion of the transferred T cell epitope in the sequence of the non-transferred protein abolishes (or decrease) transfer of the epitope, and reciprocally. An important control of HLA-pos EBV-B cells transfected with the chimeric construct, to show that the chimera is as efficiently presented endogenously as the wt sequence (for PTK2B), or not more efficiently presented as the wt protein (for PI4K2B), is indicated as (data not shown). It would be more satisfying to display these data at least in a supplementary figure.

Reply

Direct presentation data for the chimeric constructs are included in Figure 3B of the revised manuscript. Indirect antigen presentation data for the chimeric constructs are shown in Figure 3C of the revised manuscript (Figure 2B of original manuscript).

4. In figure 4, they further separate this 100,000g pellet by flotation into a density gradient, which is a good approach, following the ISEV guidelines (Lotvall J, et al. (2014). J Extracell Vesicles 3:26913.). The results show that a subtype of EVs is performing the transfer : it would be important to show, by WB, if PTK2B is present specifically in these fractions of the gradient. In addition, since they use CD9 as a marker of EVs in figure 3, can they show distribution of CD9 in the gradient: does it co-localize with the transfer activity?

Reply

We performed Western blots for CD9 and flotillin to show the distribution of EV in the gradient and included the data in Figure 8B of the revised manuscript. Unfortunately, PTK2B could not be measured in the fractions. Probably, the amount of PTK2B was below the detection limit, since only a small proportion of each fraction was used for western blotting. We used a large proportion of each fraction for loading on EBV-B acceptor cells to ensure that sufficient EV were added to measure T cell recognition.

5. Major comment for the whole article: Showing mean values of duplicate wells of one representative experiment is not satisfying. The authors must show results of at least 2 independent experiments , presented as dot plots showing the individual biological replicates, and not as bar graphs : Weissgerber TL, Milic NM, Winham SJ, Garovic VD. (2015) Beyond Bar and Line Graphs: Time for a New Data Presentation Paradigm. PLoS Biol 13(4): e1002128. If only 2 experiments have been performed for each figure,

statistical analysis is not really useful, but displaying the inter-experiment variability by using the type of representation requested in this point could be sufficient.

Reply

We changed all figures in the revised manuscript and included dot plots to show individual biological replicates.

6. The antigens used here are described as endogenous, but their transfer for presentation is observed in cells transfected to artificially express or over-express them: did the authors check by qRT-PCR that these proteins are not endogenously expressed ? If they are, what is the relative level of the transfected and endogenous protein? If the endogenous protein is not transferred (in the absence of induced over-expression), the message of natural transfer of endogenous ligands should probably be mitigated.

Reply

All antigens investigated in our study are polymorphic antigens. As such, antigen-positive donor cells and antigen-negative acceptor cells are selected based on presence and absence of the respective single nucleotide polymorphisms, respectively. In our first coculture experiments as shown in Figure 1A-C and Figure 2 in the revised manuscript, we used EBV-B cells endogenously expressing the antigens of interest as donor cells. These experiments showed intercellular transfer for endogenous antigens from PTK2B, MTHFD1 and DBY. Based on the observation that endogenous antigens can be transferred between cells, we considered the mechanism of intercellular antigen transfer as relevant and investigated the mechanism in more detail using a coculture system in which donor cells are used that are transduced with antigen-encoding genes (Figures 1D, 3-5, 7-8).

7. The article is not particularly long, and some or all supplementary figures could probably go into the main figures.

Reply

We included all supplementary figures as main figures in the revised manuscript.

General remark

Fractions collected after density gradient ultracentrifugation have been renumbered in the revised manuscript (fraction 1: highest density; fraction 12: lowest density) and are thus different from the fraction numbers in the original manuscript (fraction 1: lowest density; fraction 12: highest density). Moreover, in the original manuscript, fraction 12 was excluded

from analysis, since this fraction was not tested for T cell recognition (Fig. 4B of the original manuscript). In the revised manuscript, however, fraction 1 was included, since fractions 1-3 were pooled for Western blotting (Fig. 8B of the revised manuscript). Fraction 1 was not tested for T cell recognition (Fig. 8C of the revised manuscript).
